# The genome sequence of the sycamore, *Acronicta aceris* (Linnaeus, 1758)

**DOI:** 10.12688/wellcomeopenres.17354.1

**Published:** 2021-11-26

**Authors:** Douglas Boyes, Liam Crowley, Peter W.H. Holland, University of Oxford and Wytham Woods Genome Acquisition Lab, Darwin Tree of Life Barcoding collective, Wellcome Sanger Institute Tree of Life programme, Wellcome Sanger Institute Scientific Operations: DNA Pipelines collective, Tree of Life Core Informatics collective, Darwin Tree of Life Consortium

**Affiliations:** 1 UK Centre for Ecology & Hydrology, Wallingford, UK; 2 Department of Zoology, University of Oxford, Oxford, UK

**Keywords:** Acronicta aceris, the sycamore, genome sequence, chromosomal, Lepidoptera

## Abstract

We present a genome assembly from an individual female *Acronicta aceris *(the sycamore; Arthropoda; Insecta; Lepidoptera; Noctuidae). The genome sequence is 466 megabases in span. The complete assembly is scaffolded into 32 chromosomal pseudomolecules, with the W and Z sex chromosome assembled.

## Species taxonomy

Eukaryota; Metazoa; Ecdysozoa; Arthropoda; Hexapoda; Insecta; Pterygota; Neoptera; Endopterygota; Lepidoptera; Glossata; Ditrysia; Noctuoidea; Noctuidae; Acronictinae; Acronicta; *Acronicta aceris* (Linnaeus, 1758) (NCBI:txid987859).

## Background

*Acronicta aceris* (sycamore moth) is a widely distributed noctuid moth found in Europe, Morocco and western regions of Asia. In the UK it is locally common in southeast and central England, with a flight season from June to August. Forewing colouration of the moth varies from silvery to dark grey, with variation in ground colour likely controlled by alleles at a single locus ( Majerus, 1986). The larvae of *A. aceris* are amongst the most colourful and flamboyant of all Lepidoptera caterpillars, bearing yellow and orange hairs arranged in striking ‘punk’ tufts along the body. As the common name suggests, the larvae feed on the leaves of sycamore ( *Acer pseudoplatanus*), other maples ( *Acer* sp.) and, particularly in urban and suburban areas, horse chestnut ( *Aesculus hippocastanum*). Larvae are active from July to September and overwintering occurs as a pupa in a double-layered cocoon in bark crevices or leaf litter. It is known to occasionally overwinter as a pupa through two winters before eclosing as an imago ( Waring *et al.*, 2003). 

## Genome sequence report

**Figure 1.**
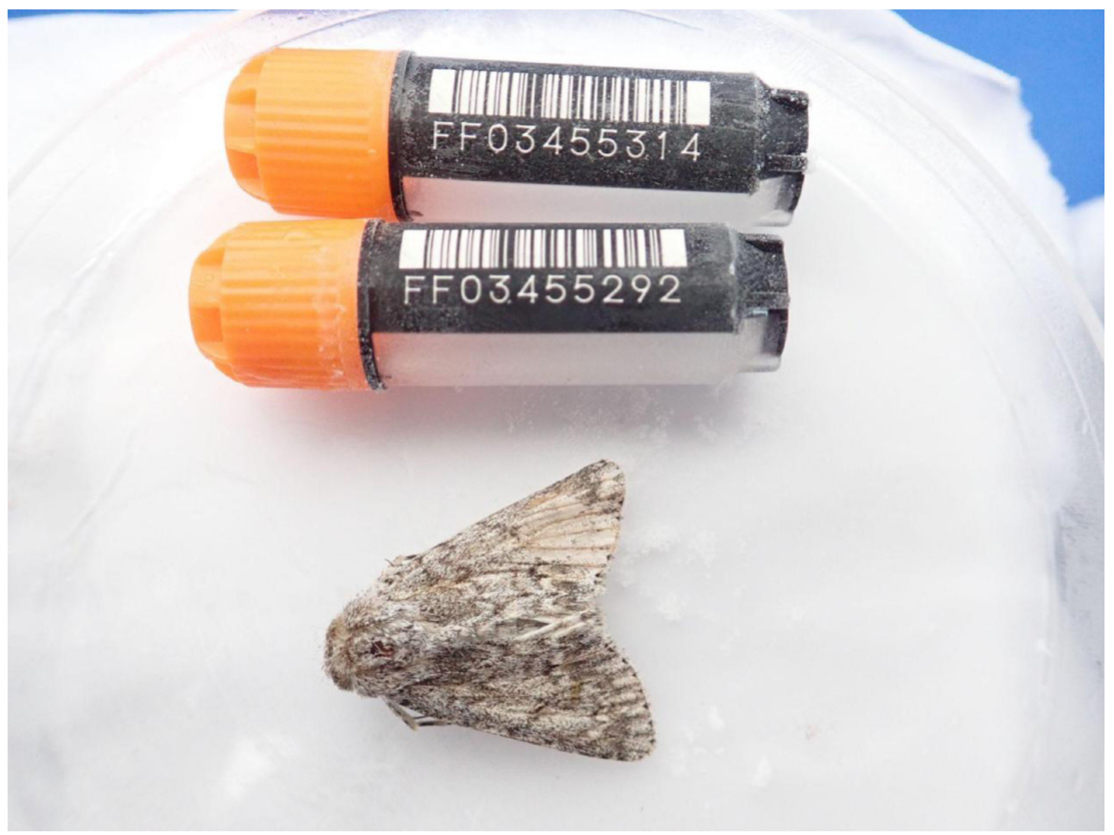
Image of the ilAcrAcer1 specimen captured prior to preservation and processing. Specimen shown next to FluidX storage tube, 43.9 mm in length.

The genome was sequenced from one female *A. berbera* ( Figure 1) collected from Wytham Woods, Oxfordshire (biological vice-county: Berkshire), UK (latitude 51.772, longitude -1.338). A total of 39-fold coverage in Pacific Biosciences single-molecule long reads and 99-fold coverage in 10X Genomics read clouds were generated. Primary assembly contigs were scaffolded with chromosome conformation Hi-C data. Manual assembly curation corrected 14 missing/misjoins, reducing the scaffold number by 20.00% and increasing the scaffold N50 by 4.33%.

**Figure 2.**
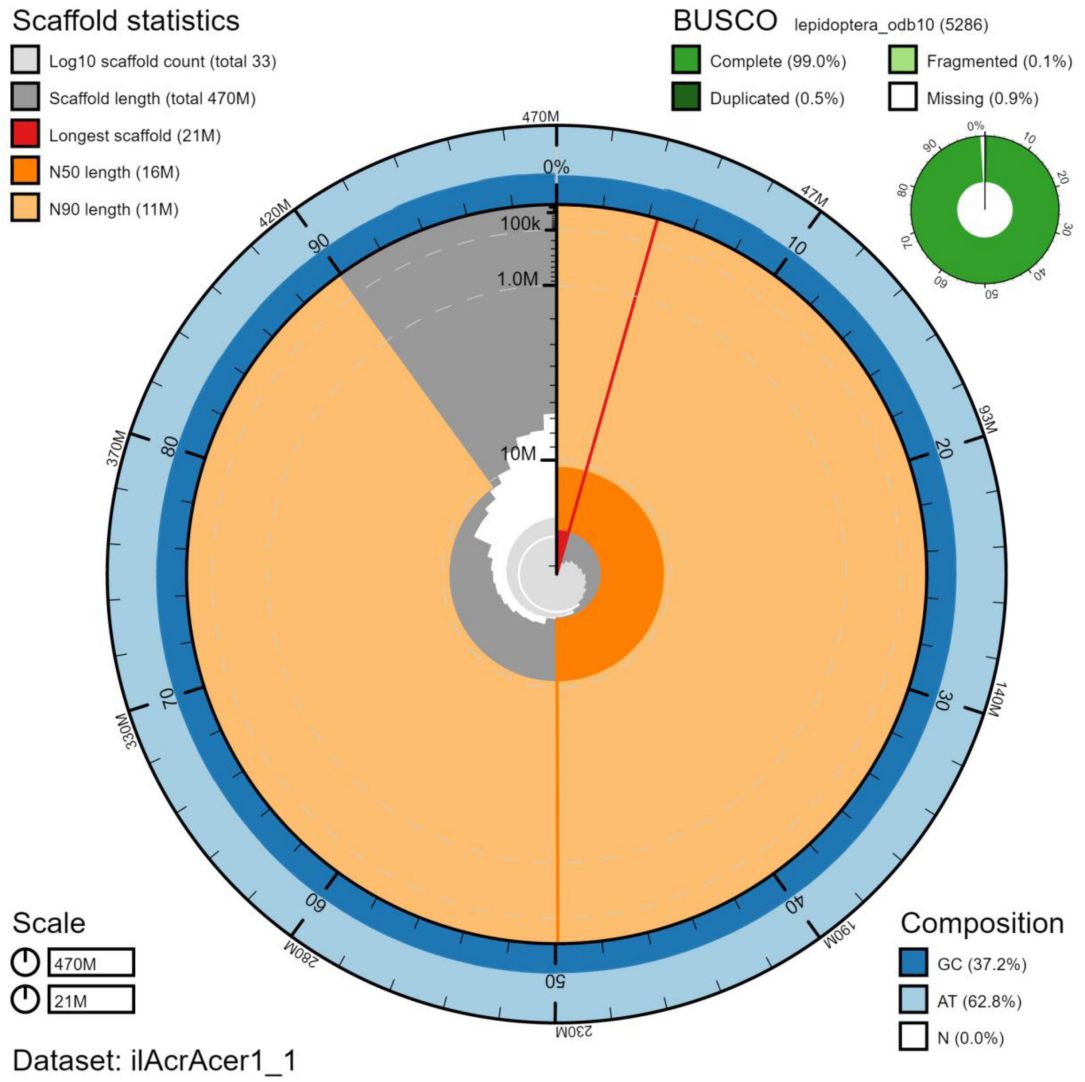
Genome assembly of *Acronicta aceris*, ilAcrAcer1.1: metrics. The BlobToolKit Snailplot shows N50 metrics and BUSCO gene completeness. The main plot is divided into 1,000 size-ordered bins around the circumference with each bin representing 0.1% of the 466,384,436 bp assembly. The distribution of chromosome lengths is shown in dark grey with the plot radius scaled to the longest chromosome present in the assembly (20,910,575 bp, shown in red). Orange and pale-orange arcs show the N50 and N90 chromosome lengths (16,061,144 and 10,539,460 bp), respectively. The pale grey spiral shows the cumulative chromosome count on a log scale with white scale lines showing successive orders of magnitude. The blue and pale-blue area around the outside of the plot shows the distribution of GC, AT and N percentages in the same bins as the inner plot. A summary of complete, fragmented, duplicated and missing BUSCO genes in the lepidoptera_odb10 set is shown in the top right. An interactive version of this figure is available at https://blobtoolkit.genomehubs.org/view/ilAcrAcer1.1/dataset/ilAcrAcer1_1/snail.

The final assembly has a total length of 466 Mb in 32 sequence scaffolds with a scaffold N50 of 16.1 Mb ( Table 1). The complete assembly sequence was assigned to 32 chromosomal-level scaffolds, representing 30 autosomes (numbered by sequence length), and the W and Z sex chromosome ( Figure 2– Figure 5; Table 2). The assembly has a BUSCO v5.1.2 ( Manni *et al.*, 2021) completeness of 99.0% (single 98.5%, duplicated 0.5%) using the lepidoptera_odb10 reference set. While not fully phased, the assembly deposited is of one haplotype. Contigs corresponding to the second haplotype have also been deposited.

**Table 1.** Genome data for *Acronicta aceris*, ilAcrAcer1.1.

*Project accession data*	
Assembly identifier	ilAcrAcer1.1
Species	*Acronicta aceris*
Specimen	ilAcrAcer1
NCBI taxonomy ID	NCBI:txid987859
BioProject	PRJEB45197
BioSample ID	SAMEA7701532
Isolate information	Female, abdomen (genome assembly), head/thorax (Hi-C)
*Raw data accessions*	
PacificBiosciences SEQUEL II	ERR6406216
10X Genomics Illumina	ERR6054961-ERR6054964
Hi-C Illumina	ERR6054960
*Genome assembly*	
Assembly accession	GCA_910591435.1
Accession of alternate haplotype	GCA_910591495.1
Span (Mb)	466
Number of contigs	45
Contig N50 length (Mb)	15.4
Number of scaffolds	32
Scaffold N50 length (Mb)	16.1
Longest scaffold (Mb)	19.0
BUSCO * genome score	C:99.0%[S:98.5%,D:0.5%],F:0.1%,M:0.9%,n:5286

*BUSCO scores based on the lepidoptera_odb10 BUSCO set using v5.1.2. C= complete [S= single copy, D=duplicated], F=fragmented, M=missing, n=number of orthologues in comparison. A full set of BUSCO scores is available at https://blobtoolkit.genomehubs.org/view/ilAcrAcer1.1/dataset/ilAcrAcer1_1/busco.

**Figure 3.**
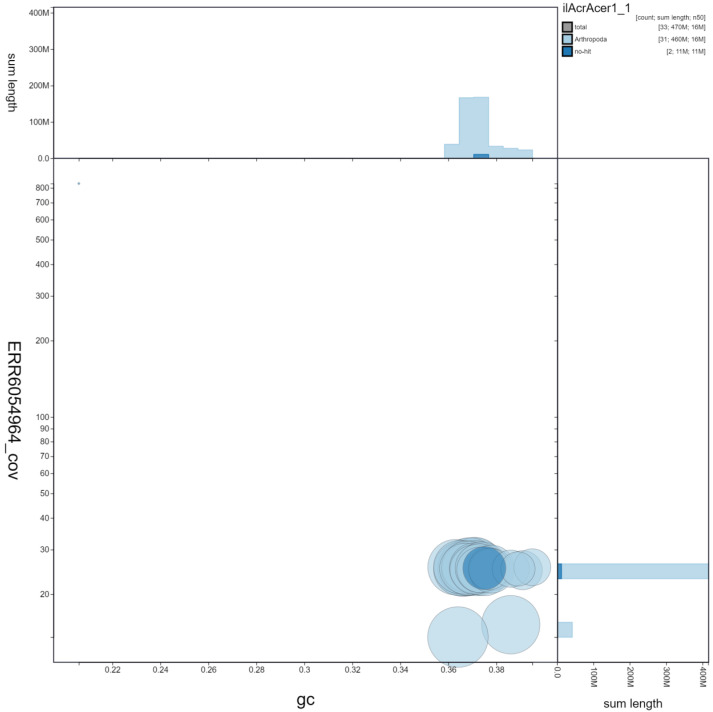
Genome assembly of *Acronicta aceris*, ilAcrAcer1.1: GC coverage. BlobToolKit GC-coverage plot. Scaffolds are coloured by phylum. Circles are sized in proportion to scaffold length Histograms show the distribution of scaffold length sum along each axis. An interactive version of this figure is available at https://blobtoolkit.genomehubs.org/view/ilAcrAcer1.1/dataset/ilAcrAcer1_1/blob.

**Figure 4.**
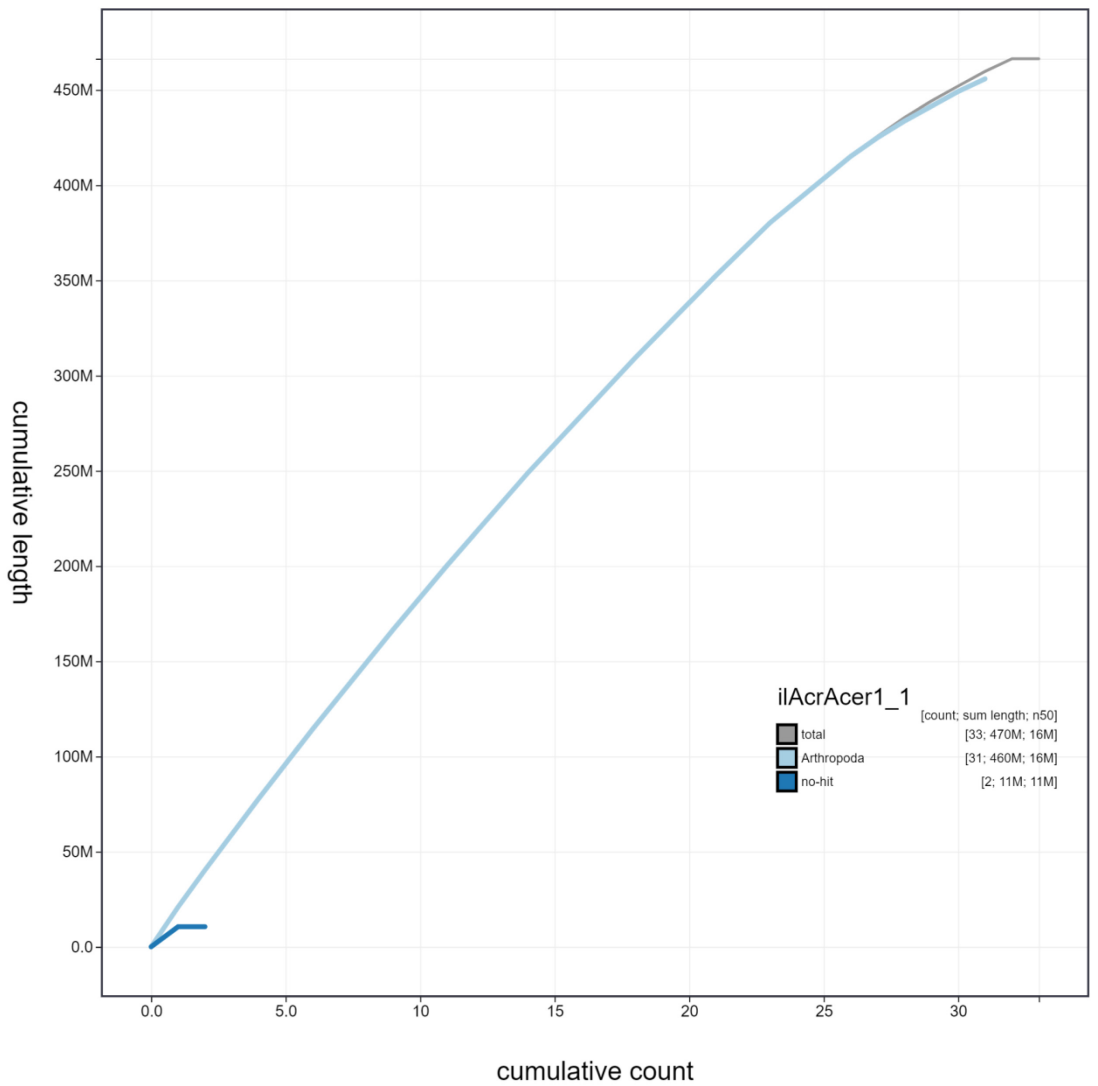
Genome assembly of *Acronicta aceris*, ilAcrAcer1.1: cumulative sequence. BlobToolKit cumulative sequence plot. The grey line shows cumulative length for all scaffolds. Coloured lines show cumulative lengths of scaffolds assigned to each phylum using the buscogenes taxrule. An interactive version of this figure is available at https://blobtoolkit.genomehubs.org/view/ilAcrAcer1.1/dataset/ilAcrAcer1_1/cumulative.

**Figure 5.**
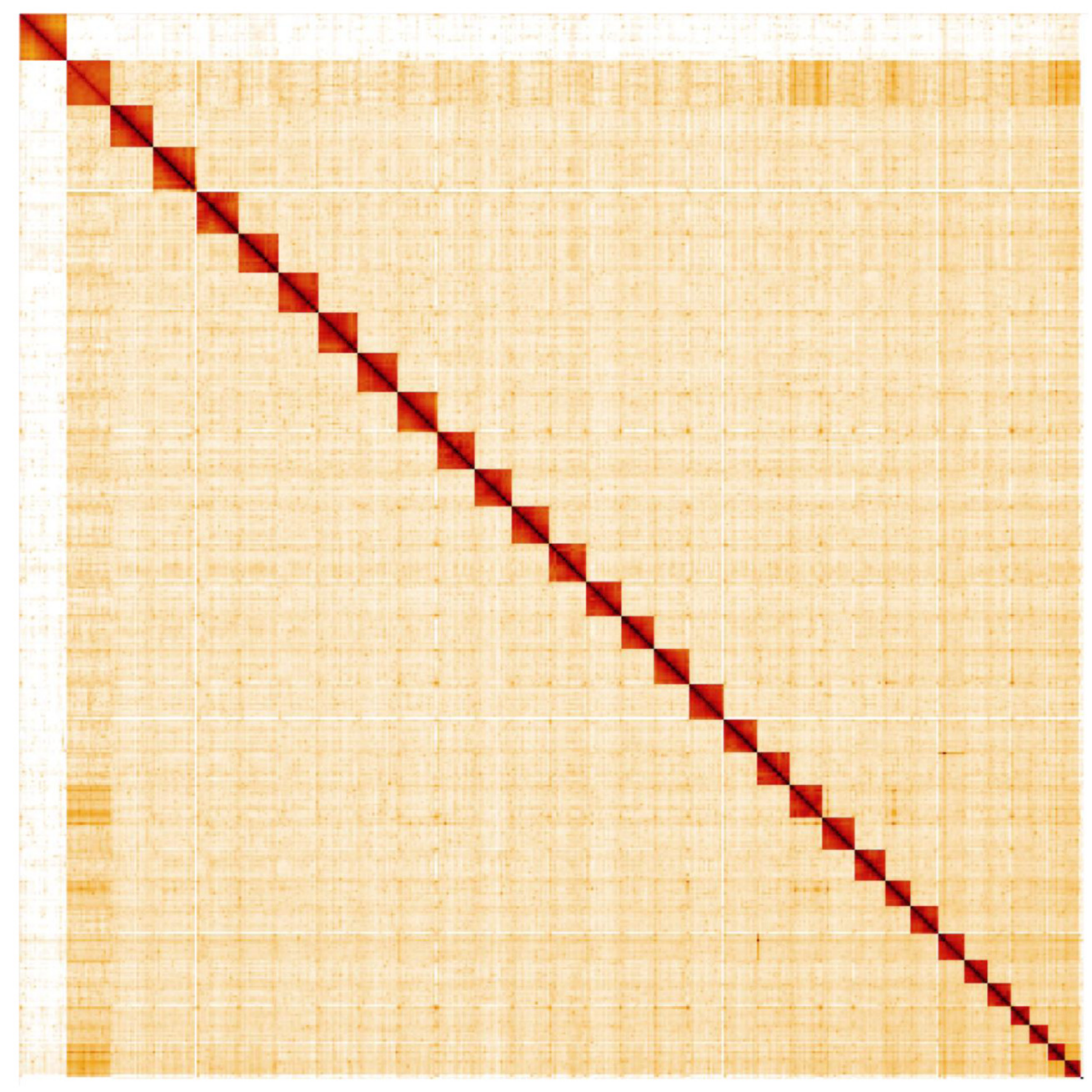
Genome assembly of *Acronicta aceris*, ilAcrAcer1.1: Hi-C contact map. Hi-C contact map of the ilAcrAcer1.1 assembly, visualised in HiGlass.

**Table 2.** Chromosomal pseudomolecules in the genome assembly of *Acronicta aceris*, ilAcrAcer1.1.

INSDC accession	Chromosome	Size (Mb)	GC%
OU342758.1	1	18.99	36.8
OU342759.1	2	18.58	37.1
OU342760.1	3	18.34	37.1
OU342761.1	4	18.02	36.9
OU342762.1	5	17.67	36.6
OU342763.1	6	17.51	36.3
OU342764.1	7	17.20	36.7
OU342765.1	8	16.99	37.2
OU342766.1	9	16.64	36.7
OU342767.1	10	16.27	36.5
OU342768.1	11	16.24	36.5
OU342769.1	12	16.06	37.1
OU342770.1	13	15.39	36.8
OU342771.1	14	15.23	36.8
OU342772.1	15	15.11	37.2
OU342773.1	16	14.79	36.7
OU342774.1	17	14.58	37.1
OU342775.1	18	14.39	37.4
OU342776.1	19	14.24	37.5
OU342777.1	20	14.04	37.1
OU342778.1	21	13.53	37.3
OU342779.1	22	11.82	37.8
OU342780.1	23	11.79	37.3
OU342781.1	24	11.31	37.8
OU342782.1	25	10.54	37.5
OU342783.1	26	9.86	37.7
OU342784.1	27	8.67	39.1
OU342785.1	28	7.85	38.6
OU342786.1	29	7.70	39.5
OU342787.1	30	6.64	38.9
OU342757.1	W	19.46	38.6
OU342756.1	Z	20.91	36.4
OU342788.1	MT	0.02	20.6

## Methods

### Sample acquisition and DNA extraction

A single female *A. aceris* (ilAcrAcer1) was collected from Wytham Woods, Oxfordshire (biological vice-county: Berkshire), UK (latitude 51.772, longitude -1.338) by Douglas Boyes, UKCEH, using a light trap. The sample was identified by the same individual, and preserved on dry ice.

DNA was extracted at the Tree of Life laboratory, Wellcome Sanger Institute. The ilAcrAcer1 sample was weighed and dissected on dry ice with tissue set aside for Hi-C sequencing. Abdomen tissue was cryogenically disrupted to a fine powder using a Covaris cryoPREP Automated Dry Pulveriser, receiving multiple impacts. Fragment size analysis of 0.01-0.5 ng of DNA was then performed using an Agilent FemtoPulse. High molecular weight (HMW) DNA was extracted using the Qiagen MagAttract HMW DNA extraction kit. Low molecular weight DNA was removed from a 200-ng aliquot of extracted DNA using 0.8X AMpure XP purification kit prior to 10X Chromium sequencing; a minimum of 50 ng DNA was submitted for 10X sequencing. HMW DNA was sheared into an average fragment size between 12–20 kb in a Megaruptor 3 system with speed setting 30. Sheared DNA was purified by solid-phase reversible immobilisation using AMPure PB beads with a 1.8X ratio of beads to sample to remove the shorter fragments and concentrate the DNA sample. The concentration of the sheared and purified DNA was assessed using a Nanodrop spectrophotometer and Qubit Fluorometer and Qubit dsDNA High Sensitivity Assay kit. Fragment size distribution was evaluated by running the sample on the FemtoPulse system.

### Sequencing

Pacific Biosciences HiFi circular consensus and 10X Genomics read cloud DNA sequencing libraries were constructed according to the manufacturers’ instructions. Sequencing was performed by the Scientific Operations core at the WSI on Pacific Biosciences SEQUEL II and Illumina HiSeq X instruments. Hi-C data were generated from abdomen tissue using the Arima v2 Hi-C kit and sequenced on an Illumina NovaSeq 6000 X instrument.

### Genome assembly

Assembly was carried out with Hifiasm ( Cheng *et al.*, 2021); haplotypic duplication was identified and removed with purge_dups ( Guan *et al.*, 2020). One round of polishing was performed by aligning 10X Genomics read data to the assembly with longranger align, calling variants with freebayes ( Garrison & Marth, 2012). The assembly was then scaffolded with Hi-C data ( Rao *et al.*, 2014) using SALSA2 ( Ghurye *et al.*, 2019). The assembly was checked for contamination and corrected using the gEVAL system ( Chow *et al.*, 2016) as described previously ( Howe *et al.*, 2021). Manual curation ( Howe *et al.*, 2021) was performed using gEVAL, HiGlass ( Kerpedjiev *et al.*, 2018) and Pretext. The mitochondrial genome was assembled using MitoHiFi ( Uliano-Silva *et al.*, 2021). The genome was analysed and BUSCO scores generated within the BlobToolKit environment ( Challis *et al.*, 2020). Table 3 contains a list of all software tool versions used, where appropriate.

**Table 3.** Software tools used.

Software tool	Version	Source
Hifiasm	0.14	Cheng *et al.*, 2021
purge_dups	1.2.3	Guan *et al.*, 2020
SALSA2	2.2	Ghurye *et al.*, 2019
longranger align	2.2.2	https://support.10xgenomics.com/genome-exome/ software/pipelines/latest/advanced/other-pipelines
freebayes	1.3.1-17-gaa2ace8	Garrison & Marth, 2012
MitoHiFi	3.0	Uliano-Silva *et al.*, 2021
gEVAL	N/A	Chow *et al.*, 2016
HiGlass	1.11.6	Kerpedjiev *et al.*, 2018
PretextView	0.2.x	https://github.com/wtsi-hpag/PretextView
BlobToolKit	2.6.2	Challis *et al.*, 2020

### Ethics/compliance issues

The materials that have contributed to this genome note have been supplied by a Darwin Tree of Life Partner. The submission of materials by a Darwin Tree of Life Partner is subject to the Darwin Tree of Life Project Sampling Code of Practice. By agreeing with and signing up to the Sampling Code of Practice, the Darwin Tree of Life Partner agrees they will meet the legal and ethical requirements and standards set out within this document in respect of all samples acquired for, and supplied to, the Darwin Tree of Life Project. Each transfer of samples is further undertaken according to a Research Collaboration Agreement or Material Transfer Agreement entered into by the Darwin Tree of Life Partner, Genome Research Limited (operating as the Wellcome Sanger Institute), and in some circumstances other Darwin Tree of Life collaborators.

## Data availability

European Nucleotide Archive: Acronicta aceris (the sycamore). Accession number PRJEB45197; https://identifiers.org/ena.embl/PRJEB45197.

The genome sequence is released openly for reuse. The *A. aceris* genome sequencing initiative is part of the Darwin Tree of Life (DToL) project. All raw sequence data and the assembly have been deposited in INSDC databases. The genome will be annotated and presented through the Ensembl pipeline at the European Bioinformatics Institute. Raw data and assembly accession identifiers are reported in Table 1.

## Author information

Members of the University of Oxford and Wytham Woods Genome Acquisition Lab are listed here: https://doi.org/10.5281/zenodo.4789929.

Members of the Darwin Tree of Life Barcoding collective are listed here: https://doi.org/10.5281/zenodo.4893704.

Members of the Wellcome Sanger Institute Tree of Life programme collective are listed here: https://doi.org/10.5281/zenodo.5377053.

Members of Wellcome Sanger Institute Scientific Operations: DNA Pipelines collective are listed here: https://doi.org/10.5281/zenodo.4790456.

Members of the Tree of Life Core Informatics collective are listed here: https://doi.org/10.5281/zenodo.5013542.

Members of the Darwin Tree of Life Consortium are listed here: https://doi.org/10.5281/zenodo.4783559.

## References

[ref-1] Challis R Richards E Rajan J : BlobToolKit - Interactive Quality Assessment of Genome Assemblies. *G3 (Bethesda).* 2020;10(4):1361–74. 10.1534/g3.119.400908 32071071 PMC7144090

[ref-2] Cheng H Concepcion GT Feng X : Haplotype-Resolved de Novo Assembly Using Phased Assembly Graphs with Hifiasm. *Nat Methods.* 2021;18(2):170–75. 10.1038/s41592-020-01056-5 33526886 PMC7961889

[ref-3] Chow W Brugger K Caccamo M : gEVAL - a web-based browser for evaluating genome assemblies. *Bioinformatics.* 2016;32(16):2508–10. 10.1093/bioinformatics/btw159 27153597 PMC4978925

[ref-4] Garrison E Marth G : Haplotype-Based Variant Detection from Short-Read Sequencing. arXiv: 1207.3907.2012. Reference Source

[ref-5] Ghurye J Rhie A Walenz BP : Integrating Hi-C Links with Assembly Graphs for Chromosome-Scale Assembly. *PLoS Comput Biol.* 2019;15(8):e1007273. 10.1371/journal.pcbi.1007273 31433799 PMC6719893

[ref-6] Guan D McCarthy SA Wood J : Identifying and Removing Haplotypic Duplication in Primary Genome Assemblies. *Bioinformatics.* 2020;36(9):2896–98. 10.1093/bioinformatics/btaa025 31971576 PMC7203741

[ref-7] Howe K Chow W Collins J : Significantly Improving the Quality of Genome Assemblies through Curation. *GigaScience.* 2021;10(1):giaa153. 10.1093/gigascience/giaa153 33420778 PMC7794651

[ref-8] Kerpedjiev P Abdennur N Lekschas F : HiGlass: Web-Based Visual Exploration and Analysis of Genome Interaction Maps. *Genome Biol.* 2018;19(1):125. 10.1186/s13059-018-1486-1 30143029 PMC6109259

[ref-9] Majerus MEN : Inheritance of Three Common Forms of Acronicta Aceris (L.)(Lepidoptera: Noctuidae). In *Proceedings and Transactions of the British Entomological and Natural History Society*.1986. Reference Source

[ref-10] Manni M Berkeley MR Seppey M : BUSCO Update: Novel and Streamlined Workflows along with Broader and Deeper Phylogenetic Coverage for Scoring of Eukaryotic, Prokaryotic, and Viral Genomes. *Mol Biol Evol.* 2021;38(10):4647–54. 10.1093/molbev/msab199 34320186 PMC8476166

[ref-11] Rao SSP Huntley MH Durand NC : A 3D Map of the Human Genome at Kilobase Resolution Reveals Principles of Chromatin Looping. *Cell.* 2014;159(7):1665–80. 10.1016/j.cell.2014.11.021 25497547 PMC5635824

[ref-12] Uliano-Silva M Nunes JGF Krasheninnikova K : marcelauliano/MitoHiFi: mitohifi_v2.0. *Zenodo.* 2021. 10.5281/zenodo.5205678

[ref-13] Waring P Townsend M Lewington R : Field Guide to the Moths of Great Britain and Ireland. British Wildlife Publishing, Hampshire.2003. Reference Source

