# Supplementary material for: The genome sequence of the sycamore, Acronicta aceris (Linnaeus, 1758)
Source: Wellcome Open Res. 2021 Nov 26;6:326. [Version 1] doi: 10.12688/wellcomeopenres.17354.1 (PMC8729028; doi:10.12688/wellcomeopenres.17354.1)
Supplement: Peer review history

# Peer review: Reviewer response for version 1

**DOI:** 10.21956/wellcomeopenres.19184.r47540

**Published:** 2021-12-24

**Authors:** Appolinaire Djikeng

**Affiliations:** 1 Centre for Tropical Livestock Genetics and Health (CTLGH), Roslin Institute, University of Edinburgh, Edinburgh, UK

The Wellcome Open Research paper by Boyes *et al.* is a genome sequencing report of the sycamore, *Acronicta aceris, *found the Southeast and central parts of England.

 The methods and strategies for sample collection (a female *A. aceris*), processing (dissection), preparation and quality control of DNA and sequencing are clearly described and appropriately referenced. Three complementary approaches including the PacificBiosciences SEQUEL II, the 10x genomics Illumina and Hi-C Illumina were used to generate high quality genome sequencing data. The data generated was further analysed very convincingly using the latest strategies for genome assembly, construction of 32 chromosomal pseudomolecules and assembly of sex chromosomes. The results are of excellent quality and add a lot more to the much-needed global genomic resources that will definitely support subsequent studies in population dynamics, distribution and the genomic changes that underpin adaptation to some environmental cues.

 I fully support this publication but wish (in addition to the comments/inputs already provided by reviewer 1) to make the following suggestions for future consideration:

1. A stronger justification for sequencing this species and the added value to the already existing other genomic resources from closely related species.
2. Under the section on data availability, it would be beneficial to steer how the data will be annotated and if there will be need to generate RNAseq data for better annotation. Additionally, I could also be useful to mention if or how within the DToL initiative to there are plans to generate additional genomic resources to increase the usefulness of this dataset. For example, these genomic resources could be further analysed and discussed in the context of understanding the genomic differences amongst devastating pests (from outside the UK).

Are sufficient details of methods and materials provided to allow replication by others?

Yes

Is the rationale for creating the dataset(s) clearly described?

Yes

Are the datasets clearly presented in a useable and accessible format?

Yes

Are the protocols appropriate and is the work technically sound?

Yes

Reviewer Expertise:

Tropical livestock genomics and genetic improvement

I confirm that I have read this submission and believe that I have an appropriate level of expertise to confirm that it is of an acceptable scientific standard.

# Peer review: Reviewer response for version 1

**DOI:** 10.21956/wellcomeopenres.19184.r47285

**Published:** 2021-12-06

**Authors:** Kumar Saurabh Singh

**Affiliations:** 1 Bioinformatics Group, Wageningen University, Wageningen, The Netherlands; 2 Plant-Microbe Interaction Group, Utrecht University, Utrecht, The Netherlands; 3 College of Life and Environmental Sciences, University of Exeter, Cornwall Campus, Cornwall, UK

Boyes et al. have sequenced a genome of *Acronicta aceris*, a Lepidopteran from *Noctuidae* family. They have assembled the genome using PacBio HiFi long-read sequencing technology and scaffolded the genome using Illumina Novaseq based on short-read data obtained from chromosome conformation capture Hi-C technology. To polish the genome high-coverage 10X genomics short-read data was also generated. Based on the adopted methodologies for genome sequencing, assembly, and quality checks, I am very much convinced that the genome is of very high quality (Contig N50:15.4 Mb; Scaffold N50: 16.1 Mb; Longest contig: 19 Mb; BUSCO C: 99%) with 32 binned chromosomes including the sex chromosomes and a mitochondrial genome. However, I have some minor comments which might improve the quality of this genome note:

1. I think a few lines of the motivation behind the sequencing of this moth is missing in the background. Particularly, I would like to see why it is important to sequence the genome of this moth and what value it will bring to the overall Lepidopteran genomics.
2. Authors have performed one round of polishing using 10X short-read data, but they have not given any information about what proportion of variants they have observed before and after polishing. This will further add knowledge to the quality of the long-reads and to the overall assembly quality.
3. I think it would be useful to the readers if the authors add an estimate of the base-level accuracy (QV) for the assembly. QV values are useful for downstream applications like population or functional genetics. This stat could be easily calculated using Merqury (Rhie *et al.* *Genome Biol* **21, **245 (2020 ^1^))

Are sufficient details of methods and materials provided to allow replication by others?

Yes

Is the rationale for creating the dataset(s) clearly described?

Yes

Are the datasets clearly presented in a useable and accessible format?

Yes

Are the protocols appropriate and is the work technically sound?

Yes

Reviewer Expertise:

Bioinformatics; Genome informatics; Integrative omics;

I confirm that I have read this submission and believe that I have an appropriate level of expertise to confirm that it is of an acceptable scientific standard.

## References

[1] : Merqury: reference-free quality, completeness, and phasing assessment for genome assemblies. *Genome Biology* .2020;21(1) : 10.1186/s13059-020-02134-9 10.1186/s13059-020-02134-9 32928274PMC7488777
